# Electrochemotherapy using thin-needle electrode improves recovery in feline nasal planum squamous cell carcinoma - a translational model

**DOI:** 10.20517/cdr.2022.24

**Published:** 2022-06-21

**Authors:** Matías Tellado, Sebastián Michinski, Joseph Impellizeri, Guillermo Marshall, Emanuela Signori, Felipe Maglietti

**Affiliations:** ^1^VetOncologia, Vet Cancer Clinic, Ciudad de Buenos Aires C1408, Argentina.; ^2^Instituto de Física del Plasma, Departamento de Física, Facultad de Ciencias Exactas y Naturales, Universidad de Buenos Aires-CONICET, Ciudad de Buenos Aires C1428, Argentina.; ^3^Veterinary Oncology Services, PLLC New York, NY 10065, USA.; ^4^Laboratory of Molecular Pathology and Experimental Oncology, Institute of Translational Pharmacology, CNR, Rome 00133, Italy.; ^5^Instituto Universitario del Hospital Italiano-CONICET, Ciudad de Buenos Aires C1199, Argentina.

**Keywords:** Cancer, SCC, nose, cat, electroporation, ECT, tumor, cSCC

## Abstract

**Aim:** Cutaneous squamous cell carcinoma (cSCC) is a common disease in patients exposed to UV-light and human papillomavirus. Electrochemotherapy, a well-established treatment modality with minimum side effects in human and veterinary medicine, circumvents chemoresistance to bleomycin by the use of electric fields. However, patients are sensitive to the trauma produced by the insertion of the needles that lengthen recovery times, particularly cats with nasal planum cSCC. To address this matter, we developed thin-needles electrodes.

**Methods:** Thin-needles electrodes developed using computer simulations and plant tissue models were compared to standard electrodes. A prospective non-randomized study recruiting 52 feline patients with nasal planum cSCC was performed. Local response, anorexia, and overall survival were evaluated.

**Results:** Computer simulations and plant model experiments showed satisfactory results with both electrodes. The patients treated with the thin-needle electrode obtained similar local response rates compared to the standard group, OR 97.3% *vs.* 80%, respectively (*P* < 0.067). Most patients in the thin-needle group resumed eating in less than 48 h, as the anorexia was significantly lower (*P* < 0.0001). Using the standard electrode, most patients took 3 to 5 days to resume normal feeding. The electric current circulating in the standard electrode was 44% higher, contributing to a longer duration of anorexia due to tissue damage. The overall survival in both groups was similar.

**Conclusion:** Electrochemotherapy using thin-needle electrodes provides equivalent local response rates and overall survival compared with standard electrodes but significantly reduced return to appetite after the treatment. These results may be useful in the development of new electrodes for human patients.

## INTRODUCTION

Cutaneous squamous cell carcinoma (cSCC) is a malignant neoplasm of keratinocytes of the skin. It is one of the most common malignancies diagnosed in humans. Older people are at an increased risk, with a ten-fold increase in patients above 70 years. People with light skin color, light-colored eyes, and light hair, who are predisposed to sunburn, are at an increased risk. In this context, UV light exposure acts as a tumor-initiating and tumor-promoting factor by inducing the formation of actinic keratosis, a precursor lesion of cSCC. The mutation of both *p*53 genes is an early event in carcinogenesis, and it is the most common mutation found, but not the only one^[1]^. An association between human papillomavirus (HPV) and cSCC has been reported^[2]^.

The similarities between human cSCC and feline cSCC are remarkable. In cats, this tumor mostly occurs in areas exposed to UV light. Particularly, areas without fur, such as the nasal planum, the pinnae, and the eyelids, are at greater risk. As the damage to the skin is cumulative, the cancer mainly affects cats older than 10 years, which according to their lifespan, is equivalent to humans around 70 years of age. Typically, a history of chronic solar exposure is present, where actinic changes lead to carcinoma *in situ* (noninvasive carcinoma confined to the epidermis, similar to the actinic keratosis in human patients), which progresses to invasive carcinoma. White-haired cats have an increased risk of developing cSCC than other hair colors as they lack the protection of melanin. More than half of the cats with nasal planum cSCC have mutations of the tumor-suppressor gene p53, indicating that a genetic predisposing factor may have an important role in the development of the disease^[3-5]^. Interestingly, HPV has been isolated from cSCC in cats, supporting that it can contribute in the same way to the disease development^[6]^. Resistance to chemotherapy and failure of combined treatments (radiotherapy plus chemotherapy) is a concern, which leads to a poor prognosis among affected patients^[7-12]^. Due to the similarities between the cSCC of cats and humans, cats provide a very good translational model for improving or developing new treatment approaches^[13,14]^.

### Diagnosis

Physical examination, including evaluation of mandibular lymph nodes, is standard. The definitive diagnosis is made with histopathology. For complete staging, the presence of metastasis in the nodes should be assessed with cytology or biopsy. Full biochemical panels, three-view thoracic radiographs, and complete blood cell counts are advised before treatment^[15]^.

### Treatment options

The preferred treatment for cSCC is surgery when possible and accepted by the owner. Wide surgical resection provides long-term control of the disease in most situations^[16]^. Another effective treatment option is radiotherapy (RT), which provides good results with mild self-limiting side effects for patients at the initial stages of the disease^[17]^. RT may also be combined with surgery to improve treatment outcomes, even with incomplete resections^[18]^. When the lesion is small, up to 0.5 cm in diameter, cryosurgery is a treatment option that may be used successfully. However, many sessions may be required for adequate control of the disease^[19]^. Similarly, photodynamic therapy (PDT) is another emerging treatment option. In one study, 90% of the treated lesions achieved a complete response, but 60% of them relapsed. As with cryotherapy, PDT may require many sessions, and it is most suitable for superficial lesions^[20]^.

There is limited evidence of the use of chemotherapy for this disease. The use of carboplatin with sterile sesame oil injected into the nasal planum showed modest results, with a complete response rate of 73% and 55% progression-free survival at one year^[21]^. The exposure of the medical staff to chemotherapy during treatment is no longer acceptable in the interest of safety, and for that reason, safety measures for handling chemotherapy are mandatory. Among effective chemotherapeutic drugs for treating cSCC, bleomycin is a good choice. Bleomycin, an antibiotic with anticancer properties, was discovered by Umezawa, who published its anticancer effects in 1966. He described its curative effect on skin cancer in dogs. The dog and mouse were the first translational models for this drug^[22]^. One of the first uses of bleomycin in human patients was in the treatment of cSCC, where the minimum effective dose was 12 cycles of 6,000 IU/m^2^. Considering the average body surface area for a human patient is around 2 m^2^, a total dose of 144,000 IU was used. The treatment could be continued after the 12th dose if good results were observed^[23]^. Other studies report using doses twice as high, achieving good local responses in cSCC, but with significant pulmonary and skin complications^[24]^. Bleomycin is non-permeant to the cell membrane, and to be internalized, it has to interact with specific membrane proteins^[25,26]^. The sensitivity of tumors to bleomycin depends on the presence of these proteins in the cell membrane, as well as the concentration of an enzyme called bleomycin hydrolase capable of degrading it^[26]^. Typically, the skin and lungs have very low concentrations of this enzyme, explaining their sensitivity to it^[27]^. The drug was approved by the FDA in 1975 for the treatment of cSCC^[28]^; however, its use steadily declined over the years due to its serious side effects when cumulative doses were high. Today, bleomycin is still used in combination with other agents for the palliative treatment of cSCC in human patients, with a cumulative dose capped at 400,000 IU^[29]^. In this context, with the discovery of electrochemotherapy by Mir *et al.* in 1997, the resistance mechanisms were overcome and the drug could once again be used with greatly reduced doses, increasing its effectiveness and reducing its side effects^[26,30]^.

### Electrochemotherapy

Electrochemotherapy (ECT) is a well-established treatment modality in veterinary medicine for treating cutaneous, subcutaneous, and mucosal tumors^[31-34]^. An aspect of ECT that sometimes is difficult to understand is how a tumor previously resistant to bleomycin can become exquisitely sensitive to it, even to the point of drastically reducing doses and still obtaining very good results.

Electroporation, depending on pulse parameters, can be irreversible, where the cells affected by the electric field die by necrosis, or reversible, where the affected cells are spared. When an electroporation-based treatment is performed, there is always an extent of reversible and irreversible electroporation coexisting^[35]^.

In reversible electroporation, cell membrane permeabilization is transient, permitting the introduction of certain molecules into the cytosol, such as bleomycin^[35]^. The combination of reversible electroporation with bleomycin or cisplatin is termed ECT. Once inside the cell, bleomycin cuts DNA strands and impedes mitosis of cells inducing mitotic cell death. The treatment displays selectivity towards dividing cells, sparing quiescent ones. Then, normal cells have time to proliferate and repair tissue defects, allowing excellent cosmetic results. For these reasons, ECT provides very good local response rates with minimum side effects^[36]^.

ECT may be used independently or in combination with surgery, especially when the surgery cannot provide clean margins. ECT may be used as a neoadjuvant, adjuvant, or concomitant therapy^[37-40]^. Some authors have reported an objective response rate of 97.3% when treating feline patients with nasal planum cSCC with minimum side effects^[41]^. ECT may also be used in combination with chemotherapy, RT, or cryosurgery for selected cases^[40]^. In the Discussion Section, we present our recommendations for performing ECT alone or combined with other therapies for treating cSCC in cats.

As mentioned above, it is reported in the literature that ECT has minor and self-limited side effects, along with very good clinical responses^[42]^. However, when treating the nasal planum in feline patients, we observed increased sensitivity to the trauma produced by the insertion of the needles. The lesions produced lead to subsequent anorexia that sometimes requires supportive treatment^[43,44]^. To evaluate a role for less trauma, our group developed two electrodes: (1) the first, labeled standard, is very similar to commonly used electrodes in standard ECT; and (2) a thin-needle electrode with needles of a reduced diameter. We evaluated electric field distribution and electroporated area to determine if the new design (2) would provide an adequate electric field distribution. A poorly designed electrode can lead to local recurrence since the tumor can be left insufficiently treated^[45]^. In the patients, we evaluated response rates, the severity of anorexia, and overall survival times to determine if the new electrode would improve the effectiveness of the treatment and the post-procedure anorexia.

## METHODS

**Figure 1:**
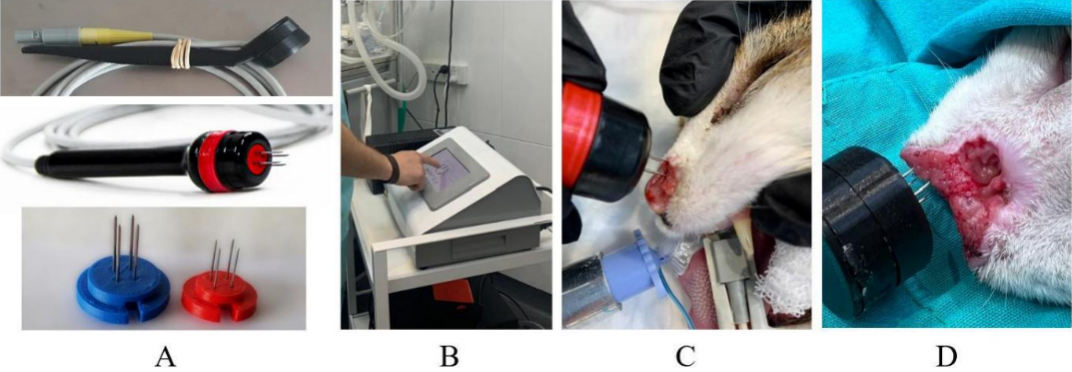
Images showing the electrodes and the electrochemotherapy procedure. (A) (top) the standard electrode; (middle) the thin-needle electrode; and (bottom) the disposables 20 G (left) and 25 G (right). (B) The operator is configuring the electroporator. (C) A patient is treated with the thin-needle electrode. (D) A patient is treated with the standard electrode.

Two electrodes were used in this study: (1) the standard electrode, composed of four 20 G needles arranged in two rows, 4 mm apart; and (2) the thin-needle electrode with the same geometry but with four 25 G needles. Both electrodes use disposable needles [Figure 1A]. The configuration of the electroporator was the same for both types of electrodes [Figure 1B].

To assess the electric field distribution, both electrodes were simulated in COMSOL Multiphysics 4.3, applying a voltage-to-distance ratio of 1000 V/cm, according to the updated standard operating procedures for ECT^[46]^.

Electrical resistance from tumors was measured using the standard electrode and used in the COMSOL model. Afterward, the design of the electrodes was validated in a simple plant model^[47]^. With the final design, the two electrodes were fabricated, and the selected patients were treated [Figure 1C and D].

### Patient selection and group allocation

Fifty-two feline patients were selected for treatment based on confirmed histopathology and nasal planum location. Cats were randomly assigned to one of the experimental groups [standard needles (1) or thin-needle group (2)] in an alternating pattern. When 15 cats were placed in the standard group (1), we added the remaining cats only to the thin-needle group (2). A full physical examination was performed, along with complete blood work (CBC, chemistry, and UA) to assess the status of the patient and the anesthetic risk. The patients were staged following the WHO criteria^[48] ^[Table 1]. In addition, feline immunodeficiency virus (FIV) was tested in all the patients and correlated with the results.

**Table 1:** Staging system for feline cSCC (WHO criteria)^[48]^

**Grade**	**Description**
T0	No evidence of tumor
Tis	Tumor *in situ*
T1	Tumor < 2 cm in diameter
T2	Tumor 2-5 cm diameter or minimally invasive
T3	Tumor > 5 cm diameter or with the invasion of subcutis
T4	Tumor invading other structures such as the fascia, muscle, or bone
N0	Absence of lymph node metastasis
N1	Presence of lymph node metastasis
M0	Absence of distant metastasis
M1	Presence of distant metastasis

As all patients were N0M0, the stages were defined as: Stage I = T1, Stage II = T2, Stage III = T3, and Stage IV = T4.

### Follow-up

Follow-up visits were scheduled every two weeks until the final response was obtained (a complete response or a lesion stable in size which was not growing back again) and monthly thereafter. We evaluated local response using modified RECIST 1.1 criteria for solid tumors^[49]^.

Complete response (CR) was defined as complete eradication of the treated tumor with re-epithelialization of skin.

Partial response (PR) was defined as a decrease of > 30% in the sum of the diameters of measurable lesions.

Stable Disease (SD) was defined as a reduction of < 30% or an increase of < 20% of the above-mentioned measurements.

Progressive disease (PD) was defined by an increase of > 20% of the above-mentioned measurements. Responses were confirmed one month after they were achieved.

Anorexia after the procedure was evaluated following the criteria of LeBlanc *et al.*^[50]^, where anorexia is defined as “a disorder characterized by a loss of appetite/decreased interest in food”. Table 2 presents the classification of anorexia.

**Table 2:** Grading of anorexia extracted from LeBlanc *et al.*^[50]^

**Grade**	**Description**
1	Complete anorexia lasting < 48 h
2	Complete anorexia lasting 2-3 days
3	Anorexia lasting 3-5 days; associated with significant weight loss (≥ 10%) or malnutrition; IV fluids, tube feeding, or force-feeding indicated
4	Life-threatening consequences; total parenteral nutrition indicated; complete anorexia lasting > 5 days
5	Death

The electric current that circulates during each pulse is shown on the screen of the electroporator. This allows us to monitor the treatment and seek a relation to the side effects.

### Anesthetic procedure

Patients were treated under general anesthesia using intramuscular administration of xylazine (Xylazine 100®, Richmond, Buenos Aires, Argentina) 0.5 mg/kg and tramadol (Tramadol®, John Martin, Buenos Aires, Argentina) 2 mg/kg. Induction was performed with intravenous administration of propofol (Propofol Gemepe®, Gemepe, Buenos Aires, Argentina) 2-3 mg/kg. For maintenance, isoflurane (Zuflax®, Richmond, Buenos Aires, Argentina) 2%-3% and intravenous fentanyl (Fentanyl Gemepe®, Gemepe, Buenos Aires, Argentina) 2 µg/kg were used. Amoxicillin with clavulanic acid (Clavamox® Zoetis, Buenos Aires, Argentina) 15 mg/kg/bid and meloxicam (Meloxivet®, John Martin, Buenos Aires, Argentina) 0.2 mg/kg/sid were administered orally for prophylaxis and analgesia after the treatment according to the needs of each patient.

### Electrochemotherapy procedure

Intravenous bleomycin was administered at 15,000 UI/m^2^ body surface area. After 5-8 min, to allow drug distribution, the electric pulses were delivered^[46]^ with a monopolar square-wave electroporator (EPV-200, BIOTEX SRL, Buenos Aires, Argentina). The pulse trains applied with both electrodes were eight 100 µs long pulses of 400 V (1000 V/cm) at a repetition frequency of 5 kHz^[46]^.

### Statistical analysis

Statistical analysis was made using MedCalc 14.8.1. To compare age, sex, and body weight, the independent samples t-test and ANOVA, respectively, were used. To compare the anorexia score, the Mann–Whitney test was used. The comparison of the response rates obtained utilized the Fisher’s exact test. For survival comparison, Kaplan–Meier curves of survival were used, and the significance was assessed with the Log-Rank test.

## RESULTS

**Figure 2:**
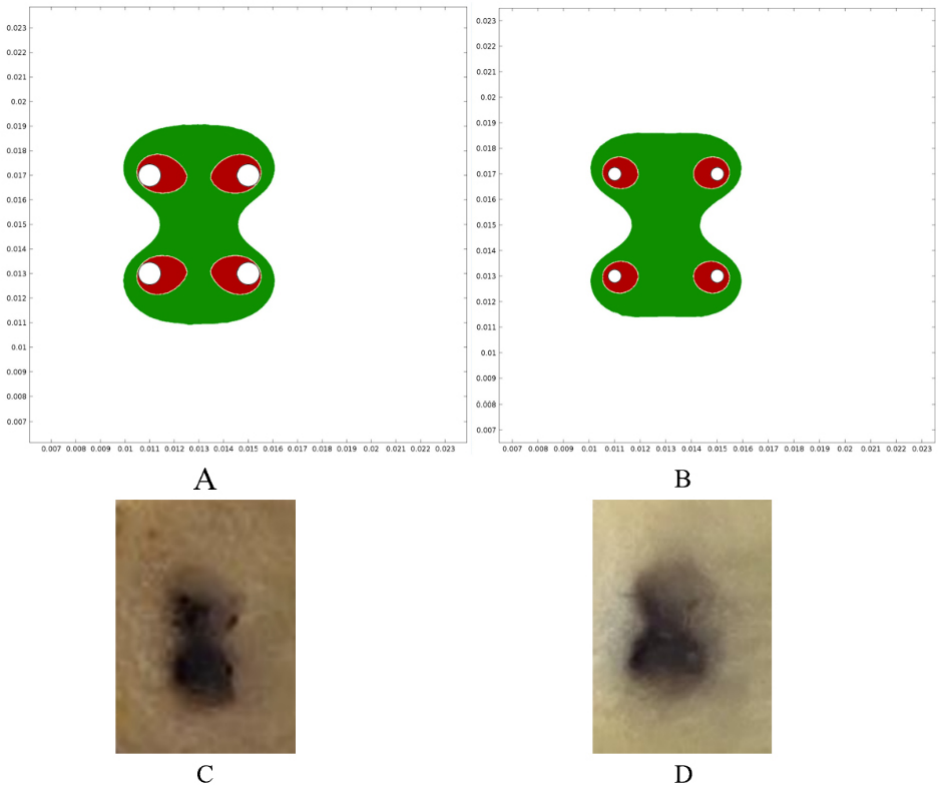
Electric field simulations and vegetable experiments. (A) Reversible and irreversible areas of permeabilization in the standard electrode. (B) The same areas in the thin-needle electrode. Green indicates the electric field was above the reversible electroporation threshold (480 V/cm) and below the irreversible electroporation threshold (1050 V/cm). Red indicates the area where the field was above the irreversible threshold (1050 V/cm). These areas were obtained using COMSOL simulations of the electric field distribution. As can be seen, in both electrodes, the region between needles is covered by reversible electroporation, but the standard electrode presents larger areas of irreversible electroporation. (C,D) The electroporated plant tissue showed an electroporated area very similar to the simulations: (C) the standard electrode (1) was used, and (D) the thin-needle electrode (2) was used.

COMSOL simulations showed that the electric field was distributed between the needles in both electrodes, which was above 480 V/cm. This electric field intensity was enough for reversibly permeabilizing the tissues. In addition, the area above 1050 V/cm was studied, which is the threshold for irreversible electroporation^[51]^. To obtain the best results, the area of reversible electroporation should be maximized (or at least cover the whole area between needles) and the area of irreversible electroporation should be kept to a minimum. This exploits the benefits of ECT, as irreversible electroporation damages the tissue without selectivity and produces necrosis that should be avoided^[52]^. As shown in Figure 2A and B, the area of reversible electroporation was adequate on both electrodes. However, the area of irreversible electroporation was larger in the standard electrode, thus contributing to the differences in trauma within this treatment group. The plant models correlated with the simulations [Figure 2C and D].

The composition of the experimental groups is shown in Table 3. There were no statistically significant differences among groups regarding age (ANOVA, *P* = 0.601), sex (independent samples *t*-test, *P* = 0.768), or body weight (ANOVA, *P* = 0.185).

**Table 3:** Composition of the experimental groups. The details of both experimental groups are presented. The differences in sex (independent samples t-test, *P* = 0.768), age (ANOVA, *P* = 0.601), and body weight (ANOVA, *P* = 0.185) were not significant between groups

	**Standard**	**Thin-needle**
n	15	37
males/females (*P* = 0.768)	7/8	16/21
FIV+	7	6
Average age [years] (*P* = 0.601)	11.3	11.1
Average body weight [kg] (*P* = 0.185)	4.78	3.92
Stage I	26.7%	43.2%
Stage II	26.7%	40.5%
Stage III	20.0%	13.5%
Stage IV	26.7%	2.7%

During follow-up, if a relapse was observed, i.e., the growth of a previously shrinking lesion, the patient was scheduled for a new treatment session. The average number of treatment sessions for the standard group (1) was 1.7 (median 2, range 1-5) and for the thin-needle group (2) was also 1.7 (median 2, range 1-3).

**Figure 3:**
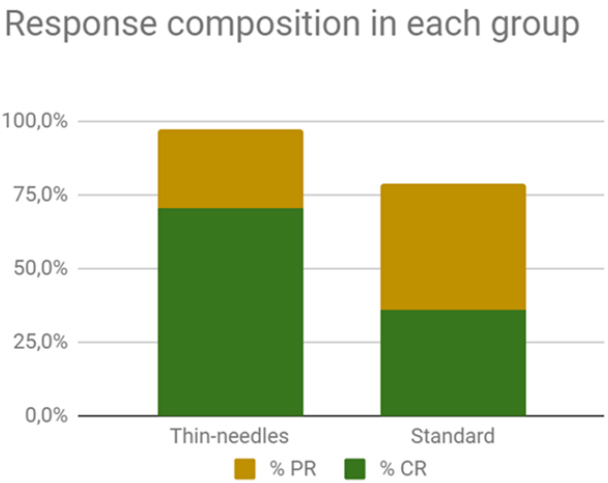
Composition of the objective response rates in each group (97.3% and 80% for the thin-needles and standard group, respectively). The differences between groups are not significant.

**Figure 4:**
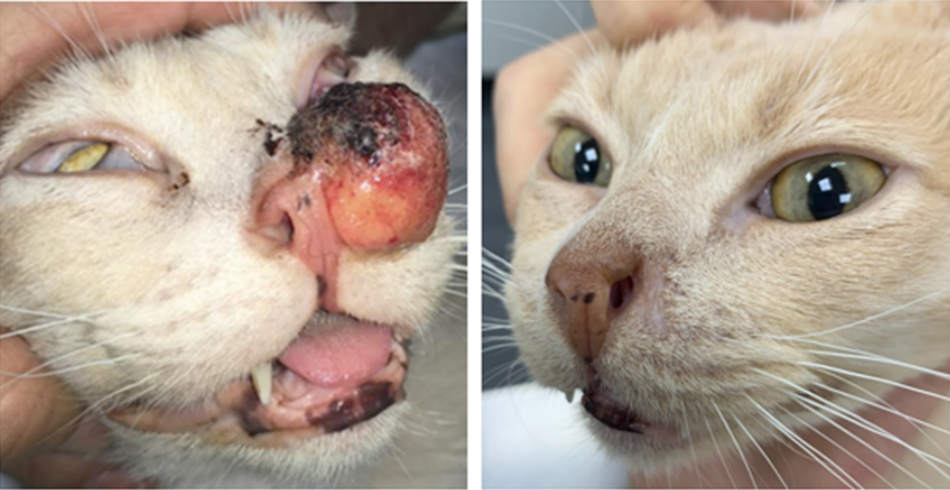
Feline patient with cSCC in the nasal planum treated with ECT using the thin-needle electrode: (left) before the treatment; and (right) 51 days after the treatment. The patient obtained CR. As can be seen, minimum tissue scarring is present where the tumor was located.

The response rate obtained in the standard group (1) was as follows: 40%CR, 40%PR, 13%SD, and no PD. The OR rate was 80%. For the thin-needle group (2), the results were 70.3%CR, 27%PR, 2.7%SD, and no PD. The OR rate was 97.3% [Figure 3]. There were no statistically significant differences between the responses obtained in the groups (Fisher’s exact test, *P* = 0.067). The cosmetic results were very good. In some cases, small scars developed not because of the treatment but because the tumor had already invaded and damaged the tissues, and thus a second intention healing with a scar is seen after the tumor responded to the treatment [Figure 4].

The anorexia score of the standard group (1) was, on average, 3.1 (median 3, range 1-5) and for the thin-needle electrode group (2) was 1.3 (median 1, range 1-3), the difference is statistically significant (*P *< 0.0001). These results show that thin needles significantly reduced post-treatment anorexia in the patients. Subjectively, the area treated looked much less inflamed after the treatment using the thin-needle electrode when compared with the standard electrode. No systemic toxicity was observed in either of the groups.

Measurements of the average electric current that circulated during the delivery of the pulses were 2.73 A in the standard group (1) and 1.89 A in the thin-needle group (2). This may be attributed to the larger surface contact with the standard needles. Approximately 44% more electric current circulated with each pulse when the standard electrode was used. The circulating electric current induces electrolysis of water molecules producing extreme pH changes in the region of the electrodes^[53,54]^. Even though they are transient, these changes can contribute to tissue damage and inflammation, and they may play a role in worsening anorexia after the treatment.

FIV+ patients showed more PR than CR when compared with the FIV- patients. This makes sense considering that ECT relies mainly on the immune system to produce a clinical response^[31]^. However, a specific study is needed to validate this observation.

**Figure 5:**
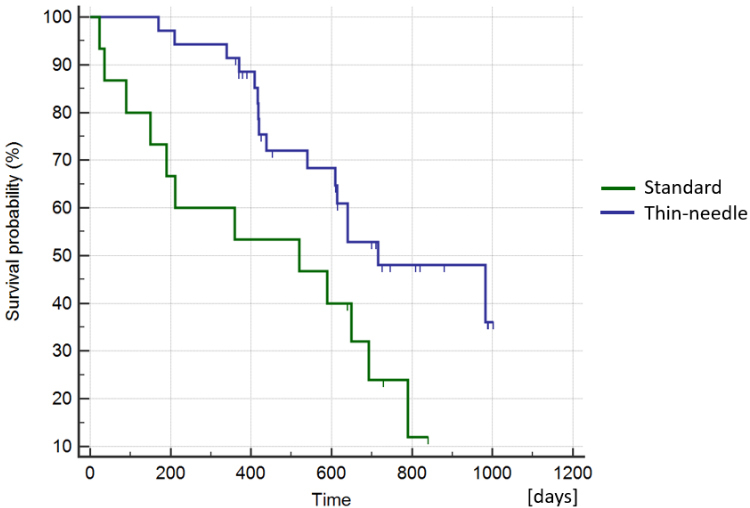
Kaplan–Meier curves of survival comparing the standard group *vs.* the thin-needle group. The curves show no statistically significant difference, meaning that the use of thin needles does not affect the long-term response (Log-Rank test, *P* = 0.019).

The median overall survival for the patients in the thin-needle group (2) was 611 days (ranging from 170-1003 days), and for the standard electrode (1) was 520 days (ranging from 23-840 days) [Figure 5]. The differences were not statistically significant, supporting that using thin needles does not reduce the survival probability of the patients (Log-Rank test, *P* = 0.019). At the end of the study, 54% (20/37) of the patients in the thin-needle electrode group (2) remained alive, while 20% (3/15) were alive in the standard electrode group (1).

**Figure 6:**
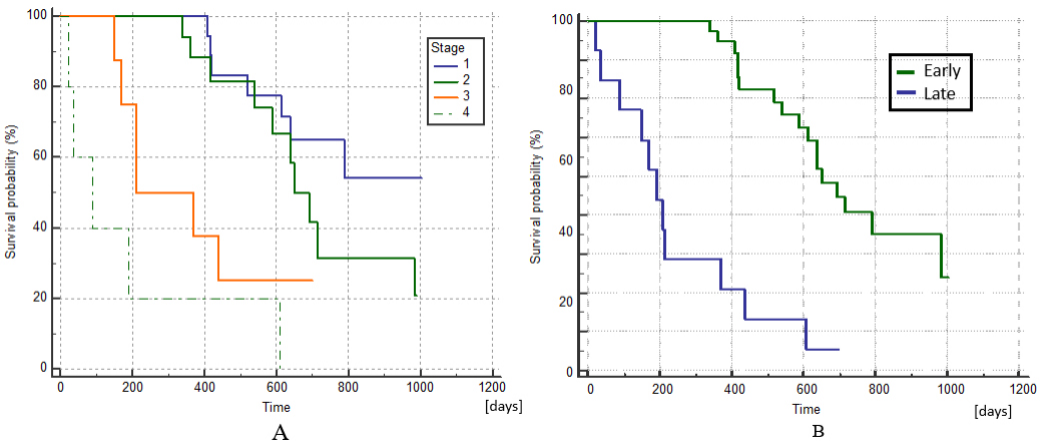
Kaplan–Meier curves of survival comparing patients at different stages of the disease. (A) There are no statistically significant differences in survival between stages I and II or between III and IV (Log-Rank test, *P* = 0.179 and *P* = 0.113, respectively). (B) Kaplan–Meier curves of survival comparing patients at early stages (I + II) with late stages (III + IV), which are significantly different (Log-Rank test, *P* < 0.0001).

The Kaplan–Meier curves of survival by stage are shown in Figure 6A, where the differences between stages I and II as well as between III and IV were not statistically significant (Log-Rank test, *P* = 0.179 and *P* = 0.113, respectively). If we group stages I and II as early stages and III and IV as late stages, survival was significantly better at earlier stages (Log-Rank test, *P* < 0.0001), as shown in Figure 6B. This result was expected and is concurrent with the experience of other authors who have used ECT^[42,43]^.

## DISCUSSION

Companion animals are increasingly accepted models of human disease^[55]^. In oncology, translation from murine models only leads to approved drugs for human use in 11% of the cases^[56]^, leading to the expenditure of billions of dollars^[57]^. The situation for companion animals as models is very different from murine models because of their unique characteristics. Companion animals work as sentinels for human diseases, due to their exposure to similar environmental hazards as their owners^[58]^. For example, the cSCC described in this work has the same risk factors and behavior as its human counterpart^[59]^, with tumors developing in the context of complex tumor–stroma interactions^[60]^ and in the presence of an intact immune system^[61]^. Both characteristics are very important for tumor resistance to different therapies present in human patients. In addition, the size of the animals allows multiple tissue sampling, and the same procedures for diagnosis and treatment are used, making the translation more efficient. The lifespan of companion animals is short; thus, clinical trials can provide results in a reduced timeframe^[55]^. Another interesting point is that owners provide very valuable information regarding their pets because they know them and can perceive changes in appetite, mood, or behavior. Finally, from the ethical viewpoint, the animals used in the studies already have the disease, and if the treatment under investigation works, there is a benefit for the patient and the owner.

ECT is a very valuable therapy in our setting, in both human and veterinary medicine. In the case of tumors of the nasal planum in cats, we use the following approach for ECT. (1) As stand alone therapy is used when the tumor can be completely treated in one treatment session. This means a tumor around 3 cm^3^, with an invasion depth of less than 2.5 cm (the length of the needles). (2) As cytoreductive therapy is used before surgery to increase the chances of success of the latter. (3) ECT may also be used during surgery, after the removal of the tumor to clean the tumor bed and reduce the risk of recurrence. In this case, plate electrodes are very useful since the tumor is removed, and the margins do not need to be too deep in the tissue. In addition, ECT may be used for treating relapses after surgery or RT. We reserve RT for very large tumors when the risk of relapse with a single ECT session is high. Very large tumors can be challenging to treat with ECT, as areas may be left untreated unintentionally^[40]^. If large tumors are meant to be treated with ECT, a close follow-up is advisable. During the follow-up, if a regrowth inside the treated area is observed, a new treatment session should be scheduled quickly. We do not perform ECT sessions on a fixed-time basis; when repeating the ECT session, a relapse is the reason after a complete remission^[42]^. In some cases, where a relapse inside the treated area is small, cryosurgery can be performed. In our experience, this is less costly for the owner than other ECT, and, for small relapses, it can be equally effective.

Human and veterinary practices of ECT share many similarities that allow us to take advantage of the results obtained from both human and veterinary patients. However, there are many differences among them, as in veterinary medicine, the variety of patients is wider, and their characteristics greatly differ. Treating the back of a Doberman, where very sharp and rigid needles are needed, is different from treating the sensitive nose of the cat. Veterinary ECT is more challenging if we consider the variety of species that can be treated, i.e., cats^[37,39,62]^, dogs^[63,64]^, horses^[65,66]^, and even wild animals^[67,68]^, and, ideally, an equal variety of electrodes should be available. The inconveniences of using the same electrodes for every patient are often accepted, but it may lead to poorer results for the patients. This challenge is not present in human medicine, since the human skin is similar among different subjects.

To address this concern and add options to the available list of electrodes for ECT, we developed a thin-needle electrode. To validate the design and study possible problems with the available electrodes, we performed simulations in COMSOL. These simulations showed that an important area of irreversible electroporation was produced when using the standard needles. This area is not negligible and should be considered in the treatment planning. Irreversible electroporation is used mainly for the treatment of visceral neoplasia, and not used in the skin, as necrosis will impact healing^[69-71]^. This delay in the healing process may have contributed to additional days of anorexia in the patients treated in the standard group. In previous work, we demonstrated the presence of extreme pH fronts emerging from the needles during the delivery of the pulses^[53]^. Even if they are rapidly neutralized by the tissue buffers, extreme pH conditions surrounding the electrodes add damage to the irreversibly electroporated area. Damaging the tumor with these extreme pH fronts may not be important, but damaging healthy tissue is. Reducing the diameter of the needles will reduce their contact surface and the circulating current (44% less electric current), thus reducing the extension of the pH fronts and thus tissue damage^[53]^. Regarding human patients, the use of thin-needle electrodes may reduce inflammation and can improve patient comfort after treatment. It may allow the reduction of medication for pain management and improve patient acceptance to return for additional treatment if needed. 

As was expected, there was no statistically significant difference between the local response rate using both electrodes (Fisher’s exact test, *P* = 0.067) or in the survival time of the patients (Log-Rank test, *P* = 0.019). This is very important since ECT is an effective treatment, and any modification to the electrodes could negatively affect its performance. Even if it was not significant, a better response rate was seen in the thin-needle group, which may be due to the higher proportion of early-stage (I + II) tumors (53.4% in the standard needle group *vs.* 83.7% in the thin-needle group).

CRs were obtained only in patients at the early stages of the disease, with the early stage a factor significantly associated with the response (Fisher’s exact test, *P* < 0.0001). Both results are in accordance with the results of other studies^[43,72]^. The microscopic bone involvement in later stages may act as a sanctuary for tumor cells^[73]^. The heterogeneity between soft tissues and bone leads to electric field inhomogeneity in the interface, reducing treatment effectiveness^[42,74]^. In addition, the bone may present with a reduced concentration of bleomycin due to its anatomical and physiological characteristics. To address this, a combination of intravenous plus intratumoral bleomycin has been proposed, allowing an optimal concentration of the drug in the poorly vascularized areas^[75,76]^.

Surgery with clean margins can provide a median survival time of 360-594 days or more^[16,77]^. Photodynamic therapy showed a median time to recurrence of 133-392 days^[78]^. Cryosurgery can provide a median disease-free time of 270 days; however, only early-stage tumors (T1 or T2) can be successfully treated^[79]^. When using ECT as a single therapy, other authors have reported an overall survival time of 210-1260 days, being the survival longer in early-stage tumors^[43,72]^. Our results with ECT are in agreement with other ECT users and seem to be comparable to surgery with clean margins. It is very encouraging, as surgery is the treatment of choice because of the unavailability of electroporation worldwide compared to surgical options. Further study is needed to compare survival times of ECT with surgery to assess if ECT can be used as first-line therapy.

**Figure 7:**
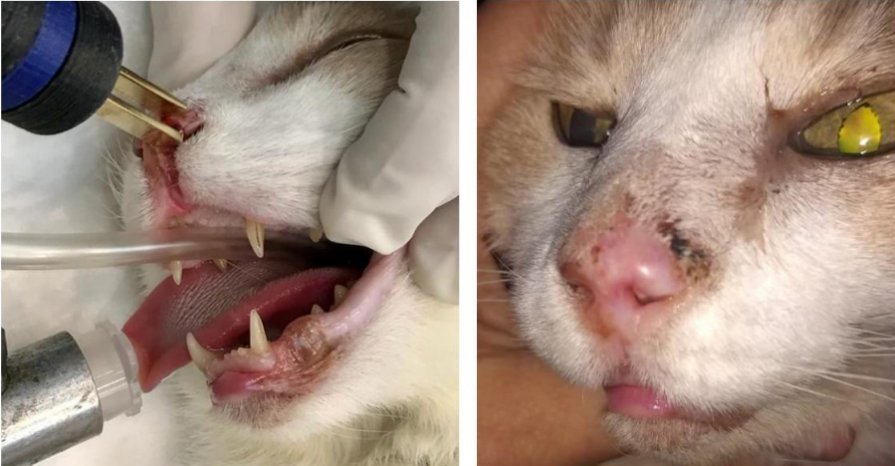
Feline patient with nasal cSCC treated with ECT using plate electrodes: (left) the patient during the treatment, where the plate electrodes were applied over the lesion; and (right) 54 days after the treatment, where a complete response was obtained.

Regarding the adverse effects, Spugnini *et al.* used an electrode similar to our standard electrode, with needles of 1 mm in diameter^[44]^. They observed electrode-induced burns and scars, and some of the patients also experienced damage to the underlying tissues. These adverse effects were resolved in 2-3 weeks. The authors used a biphasic device, and, for that reason, the contribution of the pH to the damage was lessened. Denner S. Dos Anjos *et al.* identified local side effects; particularly, hyporexia was reported with a duration of seven days, including two cases where a feeding tube had to be placed for 7-14 days after the ECT procedure^[43]^. In this case, the authors used bipolar pulses too, but plate or needle electrodes were used depending on the size of the tumor. They reported using both in many cases. However, it is not clear if the hyporexia was worse when using plates, needles, or both at the same time. Again, even if the damage related to the extreme pH changes is reduced by the use of a bipolar device, the damage attributed to the irreversible electroporation cannot be ruled out. Using both electrodes in the same area may also contribute to increasing the area of irreversible electroporation by excessive overlapping of the electric field, i.e., overtreatment. It is also worth mentioning that all the complications were adequately managed, and the patients obtained good results. Tozon *et al.* did not report local or systemic adverse effects after ECT^[72]^; in this case, plate electrodes were used, and, thus, no trauma was produced. Using plate electrodes would be an option reserved for tumors with an invasion depth of a few millimeters^[80]^. Even though we did not use plate electrodes in this work, we used them for selected cases [Figure 7]. If the tumor invades deeper, then multiple sessions are needed when using plate electrodes. For that reason, invasive tumors may be treated better using needle electrodes.

Another important aspect where human medicine diverges from veterinary medicine is the mandatory use of disposable electrodes. In the latter, only a few manufacturers offer disposable electrodes, which in the authors’ point of view, are very important for several reasons. First, new needles are sharp, and the tip is in optimal condition with each treatment. This significantly reduces the insertion trauma. Second, the conduction of the electricity is better, as there is no oxidation (which even occurs in stainless steel electrodes), providing an optimal electric field distribution. We strongly recommend using disposable needles in every case. In this study, their use was fundamental, as we kept the trauma induced by insertion to a minimum. Most patients underwent one or two treatment sessions (median 2). Spugnini *et al.* used more treatment sessions (median 4) to obtain similar objective response rates using caliper electrodes^[81]^. The fact that we needed fewer treatment sessions to achieve similar responses could be attributed to the use of disposable needles, which improve conductivity and produce a more homogeneous electric field distribution. However, in another study, Tozon *et al.*^[72]^ reported performing mostly one treatment session and an 81.8% CR rate in 11 patients. In this case, the difference is that they used plate electrodes. Denner S. Dos Anjos *et al.* mostly performed one session but used plates and needles^[43]^. The combination may overcome the use of reusable needles at the expense of longer hyporexia time due to overtreatment. In any case, further study is needed to confirm that the use of disposable needles may reduce the number of treatment sessions.

In conclusion, cSCC is a common malignancy in feline patients exposed to UV light and HPV, which shares many characteristics with its human counterpart. For that reason, they are excellent models of human disease. The treatment of choice is surgery with clean margins, as other approaches are less effective. Particularly chemotherapy is not a good option because of cSCC’s chemoresistance. However, ECT has overcome that barrier, being very effective. In particular, ECT using thin-needle electrodes in the nasal planum, when compared with standard electrodes, reduces anorexia times, improves recovery, and provides equally good results.

## DECLARATIONS

### Availability of data and materials

Data is available upon request to the corresponding author.

### Copyright

© The Author(s) 2022.
